# Cell Death Triggered by the Autophagy Inhibitory Drug 3-Methyladenine in Growing Conditions Proceeds With DNA Damage

**DOI:** 10.3389/fphar.2020.580343

**Published:** 2020-10-15

**Authors:** Javier Chicote, Víctor J. Yuste, Jacint Boix, Judit Ribas

**Affiliations:** ^1^Pharmacology of Cellular Stress Group, Department of Experimental Medicine, School of Medicine, University of Lleida, Lleida, Spain; ^2^Lleida Institute for Biomedical Research (IRBLleida), Lleida, Spain; ^3^Cell Death, Senescence and Survival Group, Department of Biochemistry and Molecular Biology, Faculty of Medicine, Autonomous University of Barcelona, Barcelona, Spain; ^4^Institute of Neurosciences, Autonomous University of Barcelona, Barcelona, Spain; ^5^Department of Experimental Medicine, Faculty of Medicine, University of Lleida, Lleida, Spain

**Keywords:** 3-methyladenine, autophagy inhibitor, basal autophagy, Ɣ-H2A.X, apoptosis

## Abstract

Macroautophagy (hereafter autophagy) is a multistep intracellular catabolic process with pleiotropic implications in cell fate. Attending to its activation, autophagy can be classified into inducible or constitutive. Constitutive, or basal autophagy, unfolds under nutrient-replete conditions to maintain the cellular homeostasis. Autophagy inhibitory drugs are powerful tools to interrogate the role of autophagy and its consequences on cell fate. However, 3-methyladenine and various of these compounds present an intrinsic capacity to trigger cell death, for instance the broadly-employed 3-methyladenine. To elucidate whether the inhibition of basal autophagy is causative of cell demise, we have employed several representative compounds acting at different phases of the autophagic process: initiation (SBI0206965 and MHY1485), nucleation (3-methyladenine, SAR405, Spautin-1 and Cpd18), and completion (Bafilomycin A_1_ and Chloroquine). These compounds inhibited the basal autophagy of MEF cultures in growing conditions. Among them, 3-methyladenine, SBI-0206965, Chloroquine, and Bafilomycin A_1_ triggered BAX- and/or BAK-dependent cytotoxicity and caspase activation. 3-methyladenine was the only compound to induce a consistent and abrupt decrease in cell viability across a series of ontologically unrelated human cell lines. 3-methyladenine-induced cytotoxicity was not driven by the inhibition of the AKT/mTOR axis. Autophagy-deficient *Fip200−/−* MEFs displayed an increased sensitivity to activate caspases and to undergo cell death in response to 3-methyladenine. The cytotoxicity induced by 3-methyladenine correlated with a massive DNA damage, as shown by *γ*-H2A.X. This genotoxicity was observed at 10 mM 3-methyladenine, the usual concentration to inhibit autophagy and was maximized in *Fip200−/−* MEFs. In sum, our results suggest that, in growing conditions, autophagy acts as a protective mechanism to diminish the intrinsic cytotoxicity of 3-methyladenine. However, when the cellular stress exerted by 3-methyladenine surpasses the protective effect of basal autophagy, caspase activation and DNA damage compromise the cell viability.

## Introduction

Macroautophagy (herein, autophagy) is a highly regulated cellular process by which cellular constituents are engulfed into autophagosomes before being degraded and reused. Autophagy is a constitutive process observed in most tissues in non-stressed nutrient-replete conditions (basal autophagy). However, it also increases in response to environmental cues such as nutrient starvation or the exposure to a plethora of stressors (inducible autophagy) (Kuma et al., 2004; Degterev et al., 2005; Mizushima, 2007; Mattiolo et al., 2015). Basal autophagy participates in essential housekeeping functions like the elimination of damaged organelles, toxic aggregates of misfolded macromolecules and, probably, in other crucial aspects of the cell and organism homeostasis (Boya et al., 2013). Nonetheless, over-stimulated autophagy may also lead to cell death type II (Clarke, 1990) due to an excessive degradation of intracellular components (Bialik et al., 2018). Regardless of the final cellular outcome, inappropriate levels of autophagy are at the origin of many pathologies, and thus modulation of this process is believed to have great therapeutic avenues. In this sense, inhibition of autophagy could be beneficial in the treatment of some types of cancer, chronic obstructive pulmonary diseases, neonatal asphyxia, and specific inflammatory-based illnesses (Galluzzi et al., 2017). Chloroquine and its derivative, hydroxychloroquine, are the only FDA-licensed drugs approved in clinical trials that aim to block autophagy. This historical shortage has been compensated by the development of an expanding arsenal of new pharmacological inhibitors. The selection of those with a better inhibitory profile and with less unspecific cytotoxicity demands of an accurate characterization.

**Figure 1:**
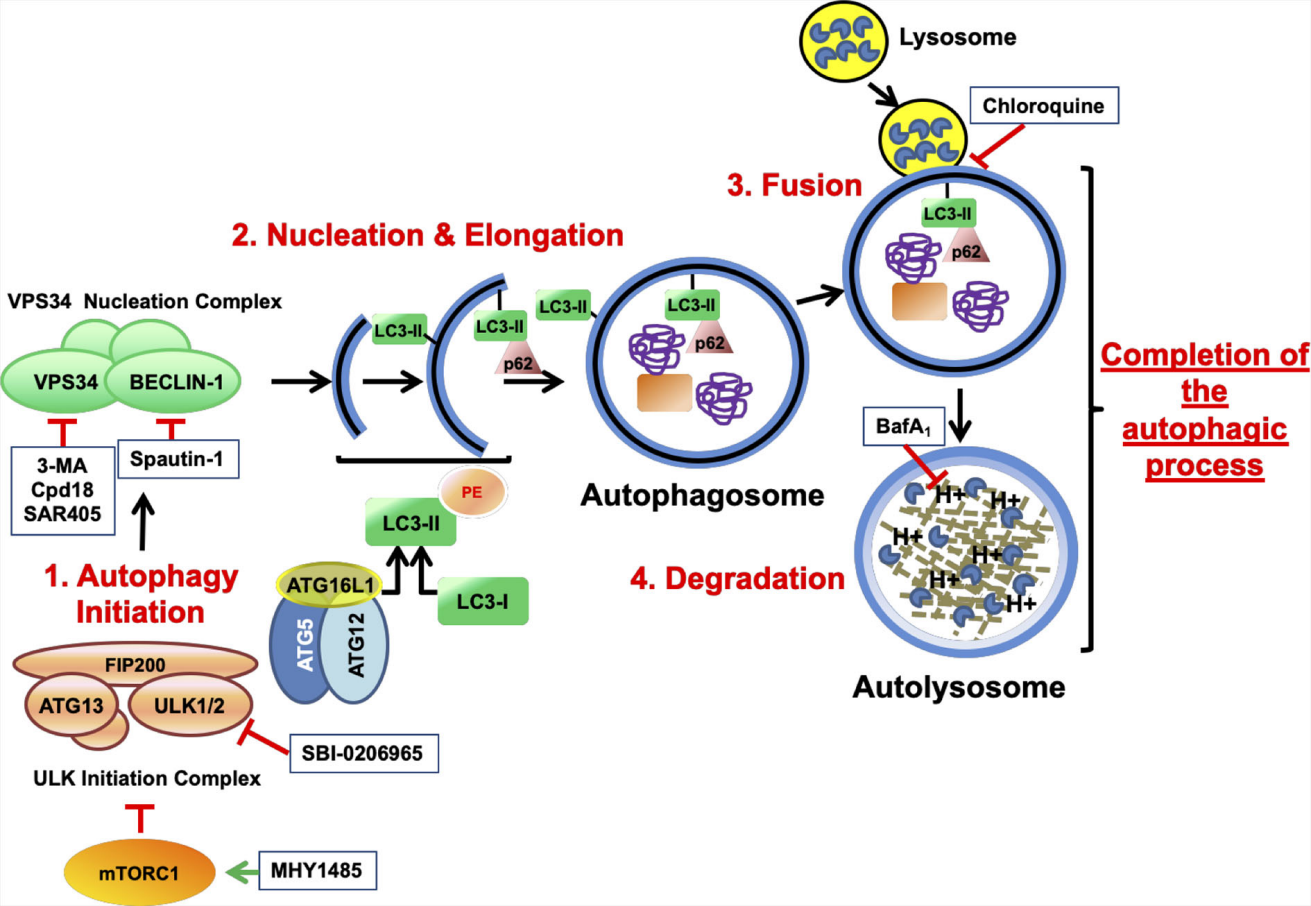
Schematic illustration depicting the process of autophagy and the targets of the autophagy inhibitory drugs. mTOR is a serine/threonine protein kinase that regulates cell growth and anabolism. Associated to other proteins, mTOR forms a complex known as mTORC1, a sensor of the nutritional state of the cell. In growing conditions, mTORC1 constitutively blocks the “ULK initiation complex” and hence, autophagy. MHY1485 is an activator of mTOR that acts through a yet unknown mechanism. The “ULK initiation complex” contains ULK1 or its homolog ULK2, FIP200, and ATG13 and triggers the first step of autophagy known as Initiation. SBI-0206965 is an inhibitor of ULK1 and ULK2. The “ULK initiation complex” drives the formation of the precursors of the autophagosomes through the direct activation of the “VPS34 Nucleation Complex”, for instance by phosphorylating VPS34 (Vacuolar Protein Sorting 34) and BECLIN-1. Spautin-1 triggers the destruction of BECLIN-1 through the inhibition of two of its deubiquitinases. On the other hand, VPS34 is a class 3 phosphatidylinositol 3-phosphate-kinase (PI3KC3) accountable for the production of the phospholipid phosphatidylinositol 3-phosphate (PI3P) necessary for the recruitment of PI3P-binding proteins that lead the nucleation and elongation of the vesicles. 3-MA, its close analog Cpd18 and SAR405 are inhibitors of VPS34. In addition, a third complex consisting of ATG16L1–ATG5–ATG12 plays a key role in the “Nucleation and Elongation” phase. This complex orchestrates a conjugation similar to what E3-ubiquitin ligases do, but in this case, they catalyze the transfer of the “ubiquitin-like” LC3-I to the lipid phosphatidylethanolamine, giving rise to LC3-II. p62/SQSTM1 is an autophagy receptor that binds the ubiquitinylated cargo and directs it to the growing double-membrane autophagosome by interacting with LC3-II and other related proteins. Finally, the “Completion of the autophagic process” includes two phases: “Fusion” and “Degradation”. During “Fusion”, the mature autophagosome fuses with lysosomes, originating a new vesicle known as autolysosome. The activation of the H^+^ pumps triggers the activation of the lysosomal hydrolases, which are in charge of degrading the cargo. Bafilomycin A_1_ inhibits the acidification of the autolysosome by blocking the vacuolar-type H^+^-V-ATPase while Chloroquine impairs the fusion of lysosomes with autophagosomes. Finally, products from degradation reach the cytosol through permeases and enter into metabolic circuitries. All drugs are depicted within squares.

Prior to the commitment into autophagy, cells must integrate information from hormonal, metabolic and stress stimuli to ensure that cell growth and proliferation are only engaged in favorable conditions. This coordination is achieved by cellular sensors like mTORC1 (mammalian target of rapamycin complex 1), which in its active form promotes cell growth and directs the metabolism into anabolic reactions. While the inhibition of mTORC1 triggers the activation of autophagy, maintaining a proper function stalls the process. Indeed, this strategy has been pharmacologically exploited by the autophagy inhibitory compound MHY1485, an activator of mTOR by a yet unknown mechanism (Choi et al., 2012). The autophagic process is orchestrated by the sequential activation of a series of protein complexes. Based on their order of participation, several autophagic phases are proposed. The “initiation phase” (**Figure 1**) involves the activation of the “ULK Initiation Complex”, which is under control of cellular sensors such as mTORC1 (D. Egan et al., 2011). This complex contains key serine/threonine kinases such as Unc-51-like kinase 1 (ULK1) or ULK2 and the scaffold protein FIP200, among other components. SBI-0206965 is an autophagy inhibitor that suppresses the kinase activity of ULK1/2 (D. F. Egan et al., 2015). The “initiation phase” gives way to the “Nucleation phase” of autophagy, which also comprises the elongation of the phagophore (**Figure 1**). This phase begins with the activation of the “VPS34 Nucleation Complex” at the sites of autophagosome formation. VPS34 (vacuolar protein sorting 34) is a class 3 phosphatidylinositol 3-kinase (PI3KC3) that forms a multiprotein complex with BECLIN-1. The generation of PI3P (phosphatidyl inositol 3-phosphate) by VPS34 and the recruitment of PI3P-binding proteins is a crucial step for the nucleation and elongation of the growing autophagosomes, which eventually seal to originate a mature vesicle (Jaber et al., 2012). 3-methyladenine has become a standard tool to test the involvement of autophagy in numerous biological paradigms through its blockage of VPS34 (Seglen and Gordon, 1982). Nonetheless, the high concentrations of 3-methyladenine required to block autophagy are facilitating its off-target effects (Wu et al., 2013). In addition, 3-methyladenine is not specific for VPS34, being class I PI3K (PI3KC1) also inhibited (Wu et al., 2010). To overcome these limitations, Cpd18 and SAR405, two inhibitors of VPS34 kinase activity with improved potency and selectivity, have been developed (Wu et al., 2013; Ronan et al., 2014; Pasquier, 2015). On the other hand, Spautin-1 inactivates VPS34 complex through the inhibition of two ubiquitin-specific peptidases (USPs) and thus increasing BECLIN-1 ubiquitination and degradation (Liu et al., 2011).

The loading of the autophagic cargo occurs during nucleation and elongation phases. It requires the participation of specific ubiquitin-like conjugation enzymes such as the E3-like complex formed by ATG16L1-ATG5-ATG12, which catalyzes the conjugation of cleaved MAP1LC3/LC3 to the lipid phosphatidylethanolamine, originating the LC3-II. LC3-II and the cargo receptor p62/SQSTM1 play relevant roles in cargo recognition and loading (Schaaf et al., 2016).

During the “Fusion phase”, the mature autophagosomes fuse to lysosomes originating the autolysosomes. These organelles contain the proteases in charge of the “Degradation” phase, whereby the cargo and structural molecules such as LC3-II and p62/SQSTM1 will be proteolyzed and recycled. For simplicity reasons, we clustered these final phases under the term “Completion phase of the autophagic process” (**Figure 1**). Chloroquine is a classical anti-malarial drug that suppresses autophagy by inhibiting the fusion of autophagosomes and lysosomes (Mauthe et al., 2018). On the other hand, Bafilomycin A_1_ belongs to the macrolide-type of antibiotics and inhibits the vacuolar H^+^-V-ATPase (V-ATPase), which is in charge of the lysosomal acidification. The pharmacological inhibition of lysosomal function allows the determination of the autophagic flux by comparing the accumulation of LC3-II and p62 in inhibited and non-inhibited conditions (Mizushima et al., 2010; Klionsky et al., 2016).

In this study, we set out to identify the autophagy inhibitory compounds that were cytotoxic in growing conditions, regardless of their autophagy inhibitory function. All the compounds tested were able to block basal autophagy but only SBI-0206965 (“Initiation phase”), 3-methyladenine (“Nucleation phase”), and Bafilomycin A_1_ and Chloroquine (“Completion phase”) triggered regulated cell death with the implication of caspases, and BAX and/or BAK. Among these drugs, 3-methyladenine showed the strongest activity at diminishing cell viability across four ontologically-unrelated human cell lines. The impairment of AKT/mTOR axis was not the leading mechanism of 3-methyladenine-driven cytotoxicity. 3-methyladenine-mediated cell death occurred independently of its basal autophagy blocking action, as demonstrated by using *Fip200−/−* MEFs. However, basal autophagy acted as a protective mechanism facing 3-methyladenine-induced caspase activation and cell death. Finally, cells cultured in the presence of cytotoxic concentrations of 3-methyladenine, displayed *γ*-H2A.X. Our study demonstrates that 3-methyladenine works as a genotoxic compound independently of its ability to block basal autophagy.

## Materials and Methods

### Cell Lines and Cell Culture Conditions

Immortalized *Bax−*/*− Bak−*/*−* (DKO) MEFs and their wild type (MEFs) counterparts were obtained from Dr. Korsmeyer’s laboratory. *Fip200−/−* MEFs and their counterparts (*Fip200+/+* MEFs) were gently supplied by Dr. Molinari and originated at Dr. Guan’s laboratory. SH-SY5Y, HeLa, HEK 293 cell lines were obtained from the American Type Culture Collection (ATCC; Manassas, VA, USA). HCT116 human adenocarcinoma cell line was kindly provided by Dr Vogelstein (The Howard Hughes Medical Institute, Sidney Kimmel Comprehensive Cancer Center, The Johns Hopkins School of Medicine, Baltimore, MD, USA).

All cell lines were maintained in DMEM (Gibco, Paisley, Scotland, UK) supplemented with 10% FCS (Gibco, Paisley, Scotland, UK) with the exception of HCT116, which were maintained in McCoy’s 5A (Biowest, Riverside, MO, USA) supplemented with 10% FCS. 5 μg/ml Plasmocin™ (InvivoGen, San Diego, CA, USA) was used as the media antibiotic. General culturing conditions were 37°C and a water-saturated, 5% CO_2_ atmosphere. Culture dishes and other plastic disposable tools were supplied by VWR (Radnor, PA, USA) and Becton Dickinson (Franklin Lakes, NJ, USA).

### Drug Treatment of Cells in Culture

As autophagy inhibitory drugs, we have used MHY1485 (Ref. 500554), Spautin-1 (Ref. 567569) and Cpd18 (Ref. 505980) from Calbiochem, part of Merck, Darmstadt, Germany. SBI-0206965 (Ref. S-7885) and SAR405 (Ref. S7682) were purchased from Selleck chemicals, Houston, TX, USA. MHY1485, SBI-0206965, SAR405 and Spautin-1 were dissolved in DMSO at a final concentration of 20 mM, 20 mM, 10 mM and 10 mM respectively. A 8 mM working solution of Cpd18 was prepared in HBSS without glucose. 3-methyladenine was obtained either from Acros Organics (Morris Planes, NJ, USA) or ApexBio (A8353, Huston, TX, USA) and dissolved in HBSS (Hank’s buffer salt solution) without glucose at a final concentration of 40 mM. Before treating the cells, 3-MA was sonicated during 20 min to prevent the formation of aggregates. Chloroquine (Sigma-Aldrich, C6628) was dissolved in PBS at a final concentration of 50 mM. Wortmannin (SC-3505) was purchased from Santa Cruz Biotechnology Inc., Dallas, Texas, USA and prepared in PBS at a final concentration of 100 mM. Bafilomycin A_1_ (Ref. 11038, Cayman chemical, Ann Arbor, MI, USA) was dissolved in DMSO at a final concentration of 1 mM. To treat cells, a 100 μM working solution was prepared in PBS. From these stock solutions, the drugs were delivered to the culture media and adjusted to the final concentrations reported in the text and figures. All the treatments were performed in the presence of ¼ HBSS + ¾ DMEM 10% FBS, the maximal concentrations of HBSS without glucose reached with the 10 mM 3-MA. Cells were also treated with this medium plus the highest concentration of DMSO resulting from the drug treatments, thus becoming the drug-untreated control of our experiments. DMSO concentrations never surpassed a 0.1% in the medium. At this concentration, we have observed no alteration in the cultured cells. To inhibit apoptotic caspases, cell cultures were treated with the pan-caspase inhibitor q-VD-OPh (Ref 551476, Calbiochem, part of Merck, Darmstadt, Germany) dissolved in DMSO and employed at the concentration indicated in the figure legend.

### Cell Staining Procedures

In order to visualize acidic vesicular organelles (AVOs), cells were stained with the monodansylcadaverine reagent (sc-214851, Santa Cruz Biotechnology Inc., Dallas, Texas, USA). In brief, cell cultures were incubated for 15 min with 50 μM monodansylcadaverine in the treatment medium before replacing it with fresh medium. To evidence the autophagic flux, DalGreen (D675, Dojindo, Japan) staining was used according to the supplier’s instructions. Briefly, 2,500 MEF cells were plated in 96-well plates. After 24 h, DALGreen reagent diluted in fresh medium was added to the cultures to reach a final concentration of 1 µM. After an incubation of 30 min, loading medium was replaced with fresh cell culture medium. Next, cells were subjected to the treatment conditions indicated in the figure. In order to detect the apoptotic nuclear morphology, cultured cells were directly stained with bis-benzimide (Hoechst 33342) at a final concentration of 2 ug/ml. Following the aforementioned procedures, cells were observed with an inverted fluorescence microscope (Nikon Eclipse Ts2R) and images captured and processed through NIS-Elements Basic Research Software. To quantify the number of monodansylcadaverine positive vesicles or the DALGreen Fluorescent intensity per cell, images of at least 50 cells from three independent wells were analyzed using ImageJ software (National Institutes of Health, Bethesda, MD, USA).

### Cell Viability Determinations

To determine cell survival, alamarBlue™ Cell Viability Assay Reagent (Pierce Biotechnology, Rockford, IL, USA) was used according to the manufacturer’s protocol. This assay couples a non-specific cellular reductase activity of viable cells to the reduction of alamarBlue (AB) into a fluorescent product. Briefly, cells plated in 96-well plates were subjected to drug treatments, then AB (1/10^th^ of the final volume) was added to each well. After an incubation of 3 h, fluorescent signal was quantified by means of a fluorescence plate reader (Infinite M200, Tecan). Percentage of viability was obtained by referring these values to the ones obtained in untreated control. To determine cell death, cells were collected by trypsinization and stained with 5 μg/ml propidium iodide (PI) from Sigma-Aldrich (St. Louis, MO, USA) directly in the cell culture medium. Following 15 min incubation at room temperature, cells were subjected to flow cytometry analysis.

### Measurement of Caspase Activation

The activity of effector caspases (DEVDase activity) was obtained by quantifying the fluorescence released from Ac-DEVD-afc substrate (Cayman chemical, Ann Arbor, MI, USA). Cells in culture were lysed and incubated in the presence of this substrate at 37°C. This procedure was initially validated by Ribas et al. (Ribas et al., 2005) and routinely used in our laboratory afterwards.

### Western-Blot

To obtain cytosolic extracts, cells were harvested and lysed in a buffer containing 50 mM NaCl, 25 mM Tris pH 6.8, 1 mM EDTA, 0.1% Triton X-100 supplemented with the Protease Inhibitor Cocktail Set III, EDTA-free (Calbiochem, part of Merck, Darmstadt, Germany). If a protein phosphorylation was examined, the phosphatase Inhibitor Cocktail II (Alfa Aesar part of Thermo Fisher scientific Inc., Kandel, Germany) was added to the buffer. Cell lysates were centrifuged at 15,000 r.p.m. for 30 min at 4°C before the determination of the protein content. To study histone H2A.X, whole cell protein extracts were obtained by lysing cells in a buffer containing 100 mM Tris/ClH pH 6.8, 1% SDS, 1 mM EDTA plus the protease and phosphatase inhibitory cocktails afore mentioned. Cell lysates were sonicated on ice for two rounds of 10 s before being clarified by centrifugation at 15,000 r.p.m. for 30 min. The amount of protein in the supernatants was quantified by a modified Lowry assay (DC protein assay, Bio-Rad, Hercules, CA, USA). Equal amounts of protein were loaded in the wells of SDS-polyacrylamide gels and separated in an electric field. Next, gels were electrotransferred onto 0.45 μm PVDF membranes (EMD Millipore, part of Merck Darmstadt, Germany) and blocked with Tris-buffered saline with 0.1% Tween 20 and 5% non-fat dry milk. Blocked membranes were probed with the following specific primary antibodies: anti-LC3B (1:1,000; L7543) from Sigma-Aldrich (St. Louis, MO, USA), anti-p62 (1:2,000; PM045) from Medical and Biological laboratories Co. “MBL” (Nagoya, Japan), anti-caspase 3 (1:2,000; clone 8G10, #9665 or 1:1,000; #9662), anti-phospho Ser757-ULK1 (1:1,000; #6888), anti-ULK1 (1:1,000; clone D8H5, #8054), anti-phospho Thr37/46-4E-BP1(1:1,000; clone 136B4, #2855), anti-4E-BP1 (1:1,000; clone 53H11, #9644) from Cell Signaling Technology, anti-*α-*Fodrin (1:15,000; MAB1622) and anti- H2A.X (1:1,000; #07-627) anti-phospho Ser139–H2A.X (1:1,000; clone JBW301, #05-636-I) from Millipore (part of Merck, Darmstadt, Germany). After incubation with the corresponding horseradish peroxidase-conjugated secondary antibody, the immunoblots were developed with the Immobilon™ reagent (Millipore, part of Merck, Darmstadt, Germany). Chemiluminescent images were recorded by means of a Chemidoc XRS apparatus and analyzed with the Image Lab version 4.0.1 software from Bio-Rad (Bio-Rad, Marnes-la-Coquette, France). Loading of each sample was assessed by staining the membranes during 5 min in a solution containing 10% methanol, 2% acetic acid, and 0.1% of Naphthol blue black Sigma-Aldrich (St. Louis, MO, USA). Then, membranes were de-stained in a 10% methanol and 2% acetic acid solution during 10 min. Membranes were allowed to dry before capturing images with the Chemidoc XRS apparatus. Otherwise stated, the westerns presented are a representative of at least three independent experiments.

To calculate the percentage of Basal Autophagic Flux (LC3-II _T+BafA1_/LC3-II _T_), LC3-II bands were detected on Chemidoc XRS (Bio-Rad, Marnes-la-Coquette, France). LC3-II protein levels were determined by computer-assisted densitometric analysis (Image Lab version 4.0.1 software from Bio-Rad). The density of each band was normalized to its respective loading control (Naphtol blue stained lane). Data obtained were expressed as the intensity ratio of LC3-II in cells subjected to an experimental condition in the presence of Bafilomycin A_1_ (“T+ BafA_1_”) to that of the same protein in cells subjected to the same experimental condition in the absence of Bafilomycin A_1_ (“T”). These intensity ratios were referred to the ratios of the basal autophagic flux in untreated cultures and expressed as a percentage, being 100% value assigned to control untreated cultures.

The intensity of the protein bands was quantified with the assistance of densitometric software (Image Lab version 4.0.1 software from Bio-Rad). After a step of normalization (see Figure legend), ratios were displayed in the figures.

## Results

### Cytotoxicity Induced by Autophagy Inhibitory Compounds Is Observed at Concentrations Inhibiting the Basal Autophagic Process

**Figure 2:**
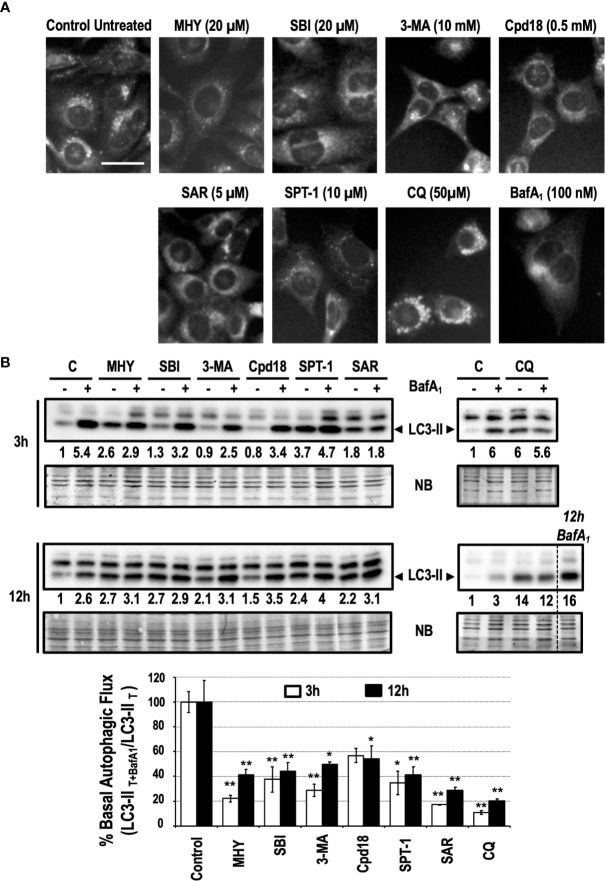
Basal autophagy is blocked in response to autophagy inhibitory drugs. **(A)** MEFs treated for 6 h with the drugs at the concentrations stated in the panel were stained with monodansylcadaverine (MDC) and observed with a fluorescence microscope. Acidic vesicles are displayed as puncta. Bar = 20 μm. Representative images of at least two independent experiments are shown. **(B)** Protein extracts of MEFs treated for 3 and 12 h, as indicated in the panel, were analyzed by western blot. “12 h BafA_1_” is a control and refers to MEFs treated for 12 h with this drug. Intensity of the LC3-II band, normalized to the loading of each lane (naphtol blue staining) and referred to “C” (untreated control) without BafA_1_, was shown. Western blots are the result of a significant experiment out of three independent experiments. A histogram representing “% Basal Autophagic Flux (LC3-II _T+BafA1_/LC3-II _T_)” at 3 h (white bars) and 12 h (black bars) of treatment was calculated as reported in the “*MATERIAL and METHODS*” section. The percentages represent the quotient between LC3-II band intensities in “T” and “T+ BafA_1_”. “T” is treatment and “T+ BafA_1_” is treatment in the presence of BafA_1_. Naphthol blue (NB) stained membrane served as a loading control. The percentage of basal autophagic flux is expressed as mean ± SEM of at least three independent experiments (n = 3). Student’s t-test *P < 0.05 and **P < 0.01.

Pharmacological inhibitors of autophagy are a common tool to interrogate the role of autophagy in a specific setting. As detailed in **Figure 1**, autophagy inhibitors acting at the initiation phase (MHY1485 -MHY- and SBI0206965 -SBI-), at the nucleation phase (3-methyladenine -3-MA-, Cpd18, Spautin-1 -SPT-1- and SAR405 -SAR-) or at the completion phase (Chloroquine -CQ- and BafilomycinA_1_ -BafA_1_-) were selected to be studied. To quickly screen the inhibitory effects of these chemicals on basal autophagy, first we employed a method to evidence the presence of acidic vesicular organelles (AVOs). MEFs subjected for 6 h to previously reported concentrations of the selected autophagy inhibitors (Choi et al., 2012; Zhao et al., 2016; Wu et al., 2010; Liu et al., 2011; Wu et al., 2013; Manic et al., 2014; Shao et al., 2014; Egan et al., 2015; Martin et al., 2018) were stained with the lysosomotropic fluorophore monodansylcadaverine (MDC). As observed in **Figure 2A**, a consistent reduction of AVOs was observed in response to the inhibitors. These results were corroborated through the quantification of MDC-positive vesicles per cell (**Supplementary Figure 1**). The accumulation of AVOs in response to CQ was a previously reported phenomenon and was employed as a positive control to evidence the maximal amount of MDC-stained vesicles (Mauthe et al., 2018). BafA_1_, which suppressed the acidic accumulation of MDC into AVOs, was employed as a negative control (Bowman et al., 1988). Next, we explored the time-dependent changes of LC3-II, a widely used read-out of the autophagic flux. Treatments consisted in incubations for 3 h or 12 h with the different inhibitors in the presence or absence of BafA_1_ for the last 3 h of treatment. Again, we confirmed that all the inhibitors blocked the basal autophagic flux to some extent (**Figure 2B**). Remarkably, at 3 h, MHY, SAR, and CQ reduced the autophagic flux percentage to less than 25% while Cpd18 reduced it to a 63% compared with the untreated control (**Figure 2B**). At 12 h, all the compounds diminished the percentage of autophagic flux to values less than 45%, with the exception of 3-MA and Cpd18, which displayed values of autophagic flux of 49 and 54%, respectively (12 h, **Figure 2B**). Of note, CQ and BafA_1,_ two common drugs used to assess the autophagic flux (Klionsky et al., 2016), triggered the greatest blockage of the autophagic flux at both 3 h and 12 h (**Figure 2B**).

**Figure 3:**
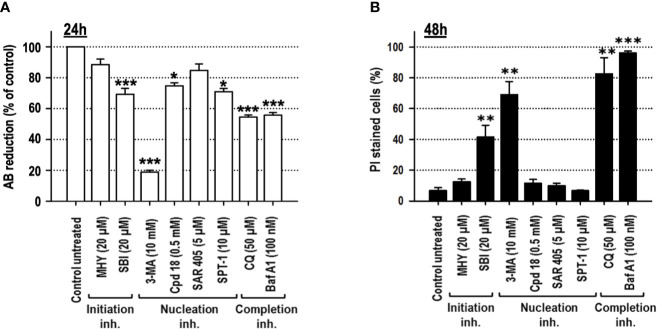
Cells cultured in growing media undergo cell death in response to the autophagy inhibitory compounds, 3-methyladenine, SBI-0206965, Bafilomycin A_1_ or Chloroquine. MEFs were treated with the drugs at the concentrations stated in the panel. **(A)** After 24 h, cell viability was measured by the cellular ability to reduce AB reagent. Bar value is the mean ± SEM (n = 3). Student’s t-test *P < 0.01, **P < 0.005 and ***P < 0.001. **(B)** After 48 h, the percentage of propidium iodide (PI)-positive cells (dead cells) was determined by flow cytometry. Bar value is the mean ± SEM (n = 3). Student’s t-test **P < 0.005 and ***P < 0.001.

Cell viability, after 24 h of treatment, was explored through the quantification of alamarBlue (AB) reduction. As shown in **Figure 3A**, among the inhibitors used, 3-MA and CQ diminished cell viability to 50% or below. Permeabilization of the external membrane with propidium iodide (PI) was evaluated at 48 h of treatment. The results evidenced that SBI (40%), 3-MA (70%), BafA_1_ (96%), and CQ (80%) triggered the most significant levels of cell lethality in growing medium, while cells challenged with MHY, SAR, Cpd18, and SPT-1 preserved the integrity of their plasma membrane (**Figure 3B**).

Altogether, despite some differences in their efficiency, MHY, SBI, 3-MA, Cpd18, SAR405, Spautin-1, CQ, and BafA_1_ are *bona fide* inhibitors of basal autophagy. Besides, our results prove that the inhibition of basal autophagy is not deleterious for MEF cultures in growing conditions.

### Cytotoxic Autophagy Inhibitory Drugs Trigger BAX- and/or BAK-Dependent Cell Death and Caspase Activation

**Figure 4:**
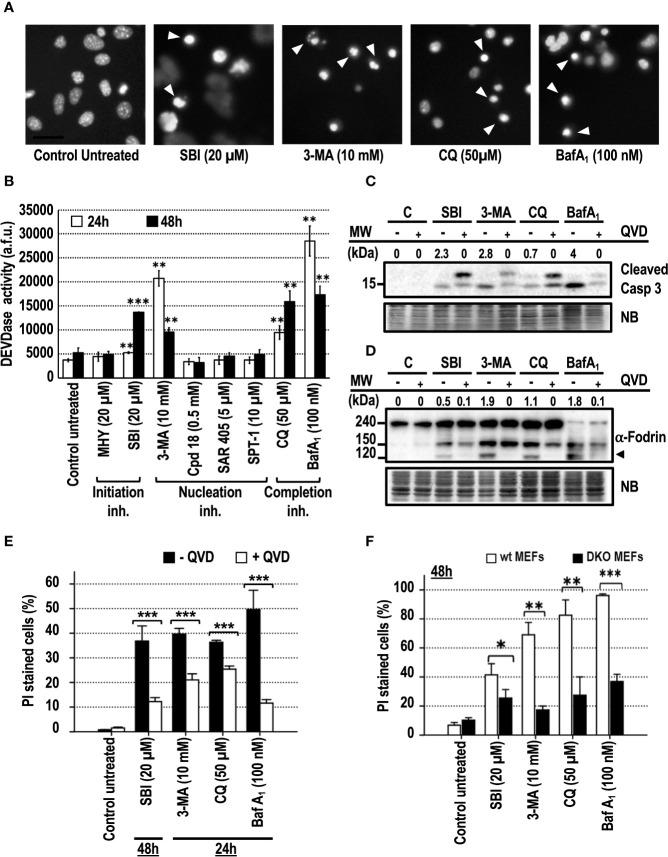
Cytotoxic inhibitors of autophagy engage the mitochondrial pathway of apoptotic cell death. MEFs were treated with the autophagy inhibitory drugs at the concentrations stated in the panel. (**A)** After 24 h of 3-MA and BafA_1_ treatment or after 48 h of SBI and CQ treatment, cells were stained with bisbenzimide 33342 and analyzed by fluorescence microscopy. Arrowheads point to the typical images of apoptotic nuclei. Bar = 40 μm. Images representative of several independent experiments are shown. **(B)** After 24 and 48 h of treatment, effector caspase activity (DEVDase activity) was quantified in arbitrary fluorescent units (a.f.u.). Bar value is the mean ± SEM (n = 3). Student’s t-test **P < 0.005 and ***P < 0.001. Protein extracts of MEFs treated with 3-MA, BafA_1_, SBI, and CQ for 48 h in the presence (+) or absence (−) of 40 μM of q-VD-OPh were analyzed by western blot. Intensity of the cleaved caspase-3 or 120 kDa *α*-Fodrin bands, normalized to the loading of each lane, was shown. Naphthol blue (NB) stained membrane served as a loading control. The images represent one representative western out of three independent experiments. The antibodies used were **(C)** anti-caspase-3 and **(D)** anti-*α*-Fodrin. **(E)** MEFs were challenged with the cytotoxic inhibitors of autophagy for the time indicated in the panel in the presence (+QVD) or absence (–QVD) of the caspase inhibitor q-VD-OPh at 40 μM. Bar value is the mean ± SEM (n = 3). Student’s t-test ***P < 0.001. **(F)** wt MEFs and *Bax*–/–*Bak*–/–MEFs (DKO MEFs) were challenged with the cytotoxic autophagy inhibitory drugs for 48 h. Drug concentrations are displayed in the panel. Cell death was quantified by means of PI incorporation and flow cytometry. Data are expressed as mean ± SEM (n = 3). Student’s t-test *P < 0.01, **P < 0.005 and ***P < 0.001.

Apoptosis is the most frequent type of regulated cell death in response to chemotherapeutical drugs. Apoptotic cell death was reported in different cell models treated for more than 24 h with 3-MA (Boya et al., 2005; Hou et al., 2012), or with BafA_1_ or CQ, used as control compounds (Boya et al., 2003; Boya et al., 2005; Walls et al., 2010; Wang et al., 2018). DNA staining of the treated cell cultures with bis-benzimide was used to highlight the presence of pyknotic and/or karyorrhectic nuclei, two of the most characteristic morphological features of apoptosis. As shown in **Figure 4A**, MEFs cultured in the presence of SBI, 3-MA, CQ, or BafA_1_ displayed pyknotic/karyorrhectic nuclear morphologies, thus pointing to apoptosis as the leading subroutine of cell death. Apoptosis is characterized by the sequential activation of caspases, resulting in a well-organized disassembly of the cell. Caspase activity was addressed at 24 and 48 h through the internal cleavage of Ac-DEVD-afc, a widely-used fluorescent substrate of caspases. Only the cell cultures subjected to SBI, 3-MA, CQ, or BafA_1_ challenges displayed a significant increase of the caspase activity with respect to the other inhibitors (**Figure 4B**). At 24 h, 3-MA, CQ, and BafA_1_ were the top activators of caspases (**Figure 4B**), while SBI required 48 h of treatment to reach equivalent levels of caspase activation. In line with these results, SBI, 3-MA, CQ, or BafA_1_ or elicited the activation of the effector caspase-3 after 48 h of treatment (**Figure 4C**). Adding the pan-inhibitor of caspases q-VD-OPh to the culture media reverted the cleavage of caspase-3 in response to the compounds mentioned before. Caspase-3 was efficiently activated in response to these inhibitors, since we observed the presence of a specific 120 kDa-cleaved fragment of *α*-Fodrin, which was avoided with q-VD-OPh (**Figure 4D**). To assess the involvement of caspases in the observed cellular toxicity, we evaluated the cell death in the presence of q-VD-OPh (**Figure 4E**). To control these experiments, q-VD-OPh was used to block cell death of cultures treated with staurosporine (STS), a canonical trigger of apoptosis. In these conditions, we confirmed a decrease of cell death from 77% (STS) to 15% (STS+q-VD-OPh, results not shown). The addition of q-VD-OPh to the cultures attenuated the cell death in response to all the autophagy inhibitory drugs tested up to 48 h of treatment (**Figure 4E** and **Supplementary Figures 2A, B**). Finally, we explored the involvement of the mitochondrial intrinsic pathway in the observed apoptotic cell death. As reported before (Boya et al., 2005), *Bax−/− Bak−/−* (DKO) MEFs were resistant against apoptotic cell death induced by CQ and BafA_1_. Cell death of DKO MEFs and wt MEFs challenged with SBI, 3-MA, CQ, or BafA_1_ was compared. As shown in **Figure 4F**, DKO MEFs were resistant against the cytotoxicity mediated by all the employed compounds, including SBI and 3-MA. Altogether, these results show that caspases and BAX and/or BAK orchestrate the cell death induced by the assayed cytotoxic autophagy inhibitory drugs. Therefore, cytotoxicity comes from a regulated intracellular signaling rather than an uncontrolled mechanism of cell death.

### Among the Autophagy Inhibitors Employed, 3-MA Is the Most Effective Compound at Decreasing Cell Viability of Several Ontologically Unrelated Human Cell Lines

**Figure 5:**
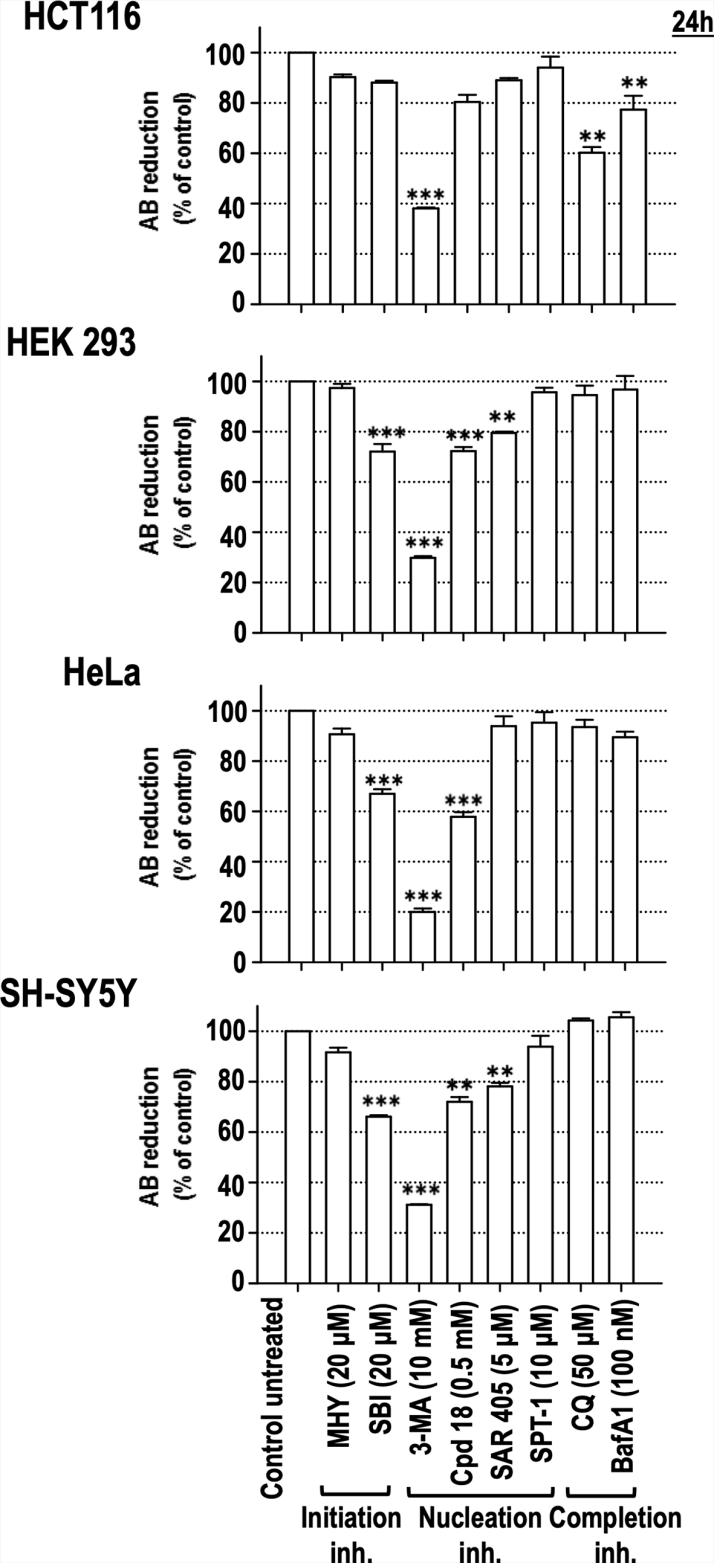
Effects of autophagy inhibitory drugs on the cellular viability in different human cell lines. HCT116, HEK293, HeLa, and SH-SY5Y cells were treated with the “Initiation” and “Nucleation” autophagy inhibitory drugs from previous experiments. Drug concentrations are indicated in the panel. Cell viability was measured by the AB reducing procedure after 24 h of treatment. Bar value is the mean ± SEM (n = 3). Student’s t-test **P < 0.005 and ***P < 0.001.

To confirm the cytotoxicity of the assayed autophagy inhibitory compounds, an ontologically different panel of human cell lines, such as HCT116 (colon adenocarcinoma), HEK293 (human embryonic kidney), HeLa (cervix adenocarcinoma), and SH-SY5Y (neuroblastoma), was challenged with the initiation, nucleation, and completion inhibitors. As shown in **Figure 5**, 3-MA decreased the viability of all the assayed cell lines to less than 40% compared to control populations. In the same line, the 3-MA derivative Cpd18 also elicited a significant reduction in cell viability in three out of four cell lines (HEK293, HeLa and SH-SY5Y cells, **Figure 5**). On the other hand, the reduction of cell viability in response to SBI was similar to the one observed with Cpd18 (**Figure 5**). Notably, at the assayed concentrations, CQ and BafA_1_ were innocuous in most of the cell lines. In sum, 3-MA emerges as the most effective autophagy inhibitor at decreasing cell viability across several unrelated cellular models.

### Basal Autophagy-Deficient Cells Are More Sensitive to 3-MA and SBI-Driven Cytotoxicity

**Figure 6:**
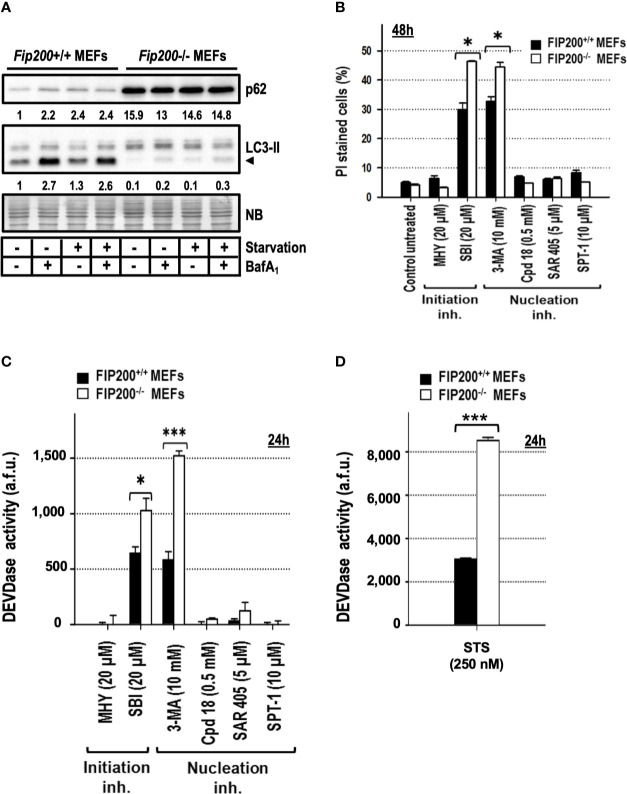
*Fip200*–/– MEFs display increased sensitivity to apoptosis triggered by 3-methyladenine or SBI-0206965, but not to MHY1485, Cpd18, SAR405 or Spautin-1. **(A)** Protein extracts from *Fip200*–/– and *Fip200*+/+ MEFs cultured in full media or Hank’s buffer without glucose (starvation) for 6h, either in the presence (+) or absence (–) of BafA_1_, were analyzed by western blot. Autophagy was evaluated by LC3-II and p62. Intensity of these bands, normalized to the loading of each lane and referred to the values of these proteins in *Fip200+/+* maintained in growing medium, was shown. Naphthol blue (NB) stained membrane served as a loading control. The image belongs to a representative image out of three independent experiments. **(B)** *Fip200*–/– and *Fip200*+/+ MEFs were challenged with the drugs at the concentrations indicated in the panel. After staining with PI, the percentage of dead cells was determined by flow cytometry. Bar value is the mean ± SEM (n = 3). Student’s t-test *P < 0.01 **(C, D)** Effector caspase activity (DEVDase activity) was quantified in arbitrary fluorescent units (a.f.u.) after 24 h of treatment with the compounds indicated in the panel. Bar value is the mean ± SEM (n = 3). Student’s t-test *P < 0.01 and ***P < 0.001.

Deletion of the focal adhesion kinase family interacting protein of 200 kDa (FIP200) avoids the formation of LC3-II, ATG16L1, and PI3P-binding protein puncta (Itakura and Mizushima, 2010) and abrogates the emergence of the autophagy isolation membrane (Kishi-Itakura et al., 2014; Tsuboyama et al., 2016). Based on these characteristics and its apical position in the autophagy pathway (**Figure 1**), it is considered as one of the best genetic approaches to suppress basal and induced autophagy. First, we assessed by western blot that *Fip200−/−* MEFs presented reduced levels of basal and inducible autophagy compared to their *wild type* genetic counterparts, *Fip200+/+* MEFs. As shown in **Figure 6A**, the generation of LC3-II was markedly reduced in *Fip200−/−* MEFs maintained in full media (“basal autophagy”, **Figure 6A**) or subjected to a complete deprivation of nutrients and trophic factors (“starvation” in **Figure 6A**). Likewise, p62 pool remained elevated in both conditions (**Figure 6A**). These results support that FIP200 is a key protein for both basal and starvation-driven autophagy. To disprove that 3-MA- or SBI-driven cytotoxicity was a consequence of the inhibition of basal autophagy, we quantified the cell death induced by these inhibitors in *Fip200+/+* and *Fip200−/−* MEFs. As negative controls, we included those non-cytotoxic compounds above-employed (MHY, Cpd18, SAR and SPT-1), which remained harmless after 48 h of incubation, regardless of the presence or the absence of FIP200 (**Figure 6B**). In contrast, cell cytotoxicity in response to 3-MA or SBI was enhanced in the autophagy-suppressed background, *Fip200−/−* MEFs (**Figure 6B**). In parallel, greater levels of caspase activity were observed in *Fip200−/−* MEFs treated with 3-MA, SBI (**Figure 6C**) or with the classical inducer of apoptosis, staurosporine (STS, **Figure 6D**). In sum, these results evidence that 3-MA- or SBI-mediated cytotoxicities are not due to the inhibition of basal autophagy. On the opposite, basal autophagy acts as a protective mechanism facing 3-MA or SBI-mediated cell death.

### 3-MA-Driven Cytotoxicity Is Shared by Its Structural Derivative Cpd18

**Figure 7:**
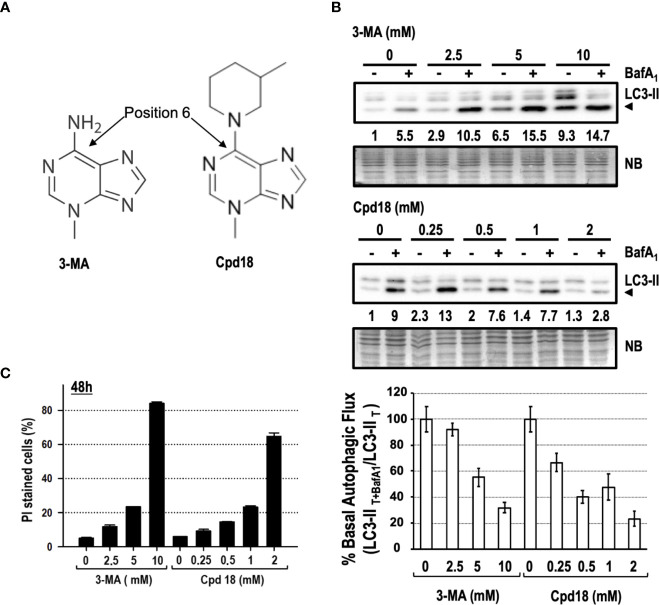
Concentration-dependent cytotoxicity of 3-methyladenine and its structural derivative Cpd18. **(A)** Chemical structure of 3-MA and Cpd18 (images borrowed from *Selleckchem* and *MerckMillipore* webpages, respectively). **(B)** Western blot of MEFs protein extracts treated for 8 h with growing concentrations of 3-MA and Cpd18 in the presence (+) or absence (–) of 100 nM BafA_1_ for the whole treatment. Intensity of the LC3-II band, normalized to the loading of each lane (naphtol blue staining) and referred to “0” without BafA_1_, was shown. Western blots are a significant experiment out of three independent experiments. A histogram representing “% Basal Autophagic Flux (LC3-II _T+BafA1_/LC3-II _T_)” was calculated as reported in the “*MATERIAL and METHODS*” section. The percentages represent the quotient between LC3-II band intensities in “T” and “T+ BafA_1_”. “T” is treatment and “T+ BafA_1_”is treatment in the presence of BafA_1_. Naphthol blue (NB) stained membrane served as a loading control. The percentage of basal autophagic flux is expressed as mean ± SEM of at least three independent experiments (n = 3). **(C)** MEFs were treated with the concentrations of 3-MA and Cpd18 indicated in the panel. After 48 h, the percentage of propidium iodide (PI)-positive cells (dead cells) was determined by flow cytometry. Bar value is the mean ± SEM (n = 3).

We focused our interest on 3-MA-elicited cell death because of its extensive use and its greater cytotoxicity compared to SBI (see **Figures 3**, **5**). 3-MA and Cpd18 are structurally related compounds that differ in a methylpiperidin group positioned at the C6 of the adenine (**Figure 7A**). We first confirmed the ability of 10 mM 3-MA and 0.5 mM Cpd18 to inhibit the autophagic flux. DALGreen is an innovative fluorescent molecule that stains the autophagosomes. Interestingly, the fluorescence of DALGreen is enhanced at acidic pH, which is suitable for monitoring the degradation phase of autophagy (Iwashita et al., 2018). After 8 h, cells treated with 3-MA (17.79 ± 0.96 a.u.f.) or Cpd18 (20.49 ± 0.94 a.u.f.) displayed a diminished DALGreen fluorescent signal compared to control populations (38.96 ± 1.08 a.u.f.), indicating that both compounds inhibit the autophagic flux (**Supplementary Figures 3A, B**). 3-MA is a widely-used drug to inhibit autophagy at concentrations ranging from 2.5 to 10 mM, being 10 mM the most frequently used (Seglen and Gordon, 1982; Petiot et al., 2000; Boya et al., 2005; Devereaux et al., 2013). Nonetheless, the calculated IC_80_ of 3-MA and its close analog Cpd18 to suppress starvation-driven autophagy are 6 and 1 mM, respectively (Wu et al., 2013). We re-evaluated the autophagy inhibitory effects of 3-MA in a concentration-dependent manner. In parallel, we also tested Cpd18 at concentrations ranging from 0.25 to 2 mM. The results with LC3-II and p62 evidenced a dose-dependent reduction of the autophagic flux in response to 3-MA and Cpd18 (**Figure 7B** and **Supplementary Figure 3B**, respectively). Next, we evaluated the cell death using the afore-employed concentrations of 3-MA and Cpd18. Cell death by 3-MA remained lower than 25% at concentrations below 10 mM but experienced an abrupt increase up to 82% at 10 mM (**Figure 7C**). The same behavior was observed at 2 mM Cpd18, which triggered a similar intensification of the cell cytotoxicity when increased from 1 up to 2 mM (23 to 64%, respectively, **Figure 7C**). Notably, the concentrations of 3-MA and Cpd18 that presented a greater attenuation of the autophagic flux were the ones associated with larger amounts of cell death. Altogether, these results stress the relevance of 3-MA and Cpd18 backbone structure to block autophagy as well as to engage cells into cell death.

### Disruption of the AKT/mTOR Axis Is Not the Main Mechanism Driving 3-MA-Mediated Cell Death

**Figure 8:**
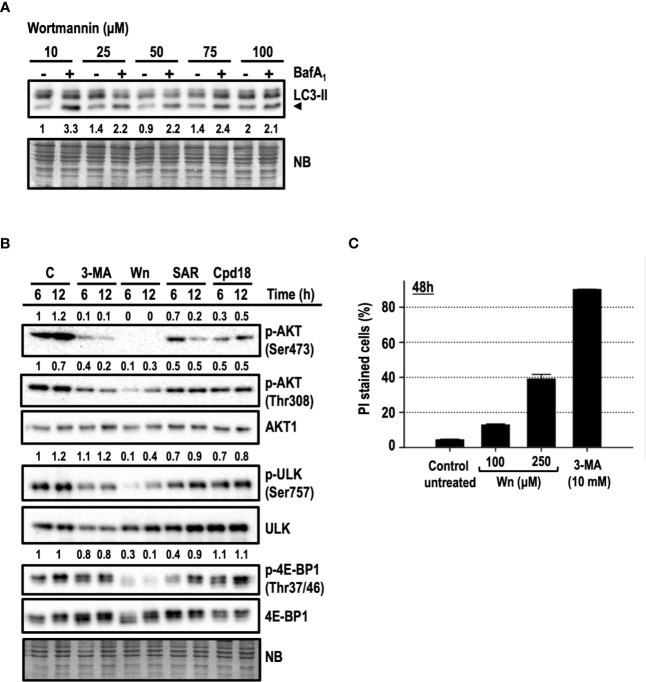
AKT/PKB and/or mTORC1 inhibition are not the leading mechanisms of 3-methyladenine-mediated cytotoxicity. **(A)** Protein extracts of MEFs subjected to growing concentrations of wortmannin either in the presence (+) or absence (–) of BafA_1_ for 8 h were analyzed by western blot. Autophagy was evaluated by LC3-II. Naphthol blue (NB) stained membrane served as a loading control. The image belongs to a representative image out of two experiments. **(B)** Protein extracts of control untreated MEFs **(C)** or MEFs treated with 10 mM 3-MA (3-MA), 100 μM Wortmannin (Wn), 5 μM SAR (SAR), 0.5 mM Cpd18 (Cpd18) at 6 and 12 h were analyzed by western blot with antibodies against phospho-AKT (p-AKT) (Ser473 and 308), AKT1, p-ULK (Ser757), ULK, p-4E-BP1(Thr37/46), and 4E-BP1. Quantifications are the ratios between the intensity of phosphorylated proteins normalized to unphosphorylated proteins. Naphthol blue (NB) stained membrane served as a loading control. The images belong to a representative experiment out of three independent repetitions. **(C)** MEFs were treated for 48 h with the drugs at concentrations stated in the panel. The percentage of propidium iodide (PI)-positive cells (dead cells) was determined by flow cytometry. Bar value is the mean ± SEM (n = 3).

In addition to its well-known inhibition of PI3K of class 3 (PI3KC3), 3-MA can also inhibit PI3K class 1 (PI3KC1) and mTOR (Ito et al., 2007; Wu et al., 2010). This lack of selectivity is also shared by wortmannin (Wn), an irreversible and potent pan-inhibitor of PI3Ks, including VPS34 (a PI3KC3) and mTOR (Arcaro and Wymann, 1993; Brunn et al., 1996). First, we assessed the blockage of the autophagic flux by wortmannin in a concentration-dependent manner. Among the concentrations employed, 100 μM wortmannin was the one exhibiting a most robust blockage of the autophagic flux (**Figure 8A**) and, hence, this concentration was selected to perform the following experiments. The serine/threonine kinase AKT is a target of phosphoinositide-dependent protein kinase-1 (PDK1), which is in turn regulated by PI3KC1 and holds a pro-survival/anti-apoptotic role in response to growth or survival factors. The activation of AKT is directed by two phosphorylations: one in threonine 308 (Thr308) by PDK1 and the other on serine 473 (Ser473) by several kinases, among them, the complex 2 of mTOR (mTORC2). We confirmed by Western blot that 10 mM 3-MA and 100 μM wortmannin were reducing the phosphorylations of AKT at Thr308 and at Ser473 already at 6 h of treatment (**Figure 8B**). Wortmannin triggered a severe abrogation of Ser473 phosphorylation and a partial, but intense, suppression of Thr308. Meanwhile, 3-MA was diminishing the phosphorylation of both positions, with a more pronounced inhibitory effect on Ser473. These results indicate that wortmannin is more efficient than 3-MA in inhibiting the activity of AKT, at least at the employed concentrations. Our experiments included Cpd18 and SAR405, which were reported to inhibit VPS34/PI3KC3 without affecting PI3KC1 (Wu et al., 2013; Ronan et al., 2014; Pasquier, 2015). As shown in **Figure 8B**, we observed a reduction of both AKT phosphorylations in response to these inhibitors. Nonetheless, the effects of SAR405 and Cpd18 were less pronounced than the effects of 3-MA.

The phosphorylations of ULK (Ser757) and 4E-BP1 (Thr37/46) are two surrogate markers of mTORC1 activity (Gingras et al., 1999; Kim et al., 2011). Wortmannin efficiently prevented the phosphorylation of ULK and 4E-BP1 at times as early as 6 h of treatment (**Figure 8B**). However, we were unable to observe a reduction of p-ULK in response to 3-MA. Likewise, 3-MA triggered only a mild reduction of p-4E-BP1. Overall, these results supported that differently from wortmannin, 3-MA was a weak inhibitor of AKT and mTORC1 activity.

To further disprove the inhibition of PI3KC1 and/or mTOR as the main mechanisms of 3-MA-driven cytotoxicity, we compared cell death in response to wortmannin or 3-MA. Despite using high concentrations of wortmannin (100 μM), cell death induced by 10 mM 3-MA was greater than the one in response to wortmannin (15% cell death, **Figure 8C**). Moreover, concentrations of wortmannin as high as 250 µM were still less toxic (40% cell death by PI, **Figure 8C**) than 10 mM 3-MA (80% cell death by PI, **Figure 8C**). In conclusion, while the inhibition of PI3KC1 and/or mTOR can partially contribute to 3-MA-mediated cell death, we rule out these phenomena as the main mechanisms guiding the 3-MA-elicited apoptotic cell death.

### 3-MA Induces γ-H2A.X in Cells at Cytotoxic Concentrations

**Figure 9:**
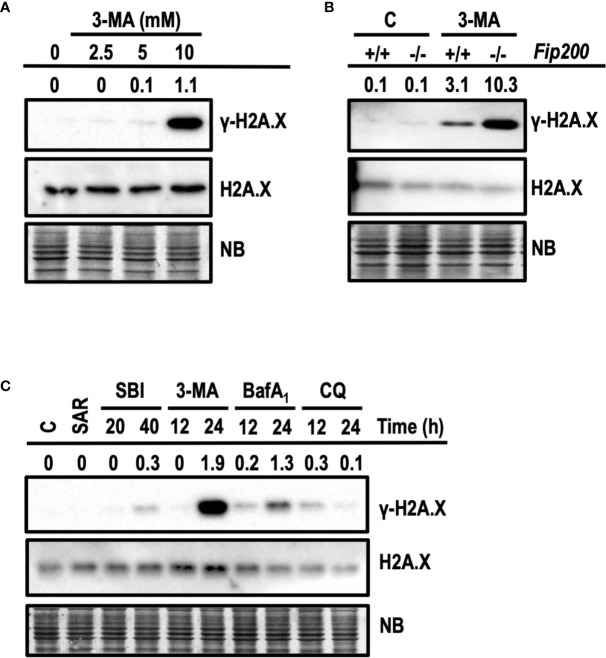
3-methyladenine triggers the phosphorylation of H2A.X at Ser139. Western blots probed with antibodies against phosphorylated Ser139-H2A.X (*γ*-H2A.X) and total H2A.X are shown. Quantifications are the ratios between the intensity of *γ*-H2A.X normalized to total H2A.X. Naphthol blue (NB) stained membrane served as a loading control. **(A)** Protein extracts of wt MEFs treated with 3-MA at 2.5, 5, and 10 mM for 24 h. Extracts of control cells are depicted as “0”. **(B)** Protein extracts of *Fip200*–*/*– and *Fip200+/+* MEFs challenged during 24 h with 10 mM 3-MA or left untreated **(C)** Protein extracts of MEFs untreated **(C)** or treated for 24 h with 5 μM SAR, for 20 and 40 h with 20 μM SBI and for 12 and 24 h with 10 mM 3-MA, 100 nM BafA_1_ and 50 μM CQ. Images in **(A–C)** are a representative experiment out of, at least, three independent experiments.

Next, we interrogated whether apoptotic concentrations of 3-MA were also damaging the DNA. DNA damage was evaluated by the presence of the phosphorylated histone H2A.X at Ser139, also known as *γ*-H2A.X (Rogakou et al., 1998). First, we evaluated the levels of *γ*-H2A.X in response to growing concentrations of 3-MA. As shown in **Figure 9A**, 10 mM of 3-MA promoted a prominent increase of *γ*-H2A.X not paralleled by non-cytotoxic concentrations of 3-MA (2.5 and 5 mM). These results indicated a relationship between the degree of cytotoxicity and the amounts of *γ*-H2A.X. To further support this observation, we explored the induction of *γ*-H2A.X in *Fip200−/−* MEFs, which were more sensitive to cytotoxicity driven by 10 mM 3-MA (**Figure 6B**). As depicted in **Figure 9B**, 3-MA-treated *Fip200−/−* MEFs displayed greater amounts of *γ*-H2A.X as compared to *Fip200+/+* MEFs. To test if the observed DNA damage is a response restricted to 3-MA, we employed the cytotoxic inhibitors studied before. As a control, we also included SAR, a non-cytotoxic inhibitor of autophagy. As shown in **Figure 9C**, increased levels of *γ*-H2A.X were evidenced in response to all of the cytotoxic inhibitors. Remarkably, 3-MA stood as the top inducer of *γ*-H2A.X among the rest of cytotoxic compounds. On the other hand, SAR was unable to promote *γ*-H2A.X. These results discard, first, the direct inhibition of VPS34 and second, the suppression of basal autophagy, as the mechanisms accountable for the 3-MA-mediated increase of H2A.X phosphorylation at Ser139. As far as we know, this is the first time that the cytotoxic inhibitors of autophagy, particularly 3-MA, emerge as inducers of DNA damage, as detected by *γ*-H2A.X.

## Discussion

The pharmacological inhibition of autophagy, either alone or in combination with other chemotherapeutical drugs, is under investigation to become a fully accepted antitumoral strategy. In this sense, the therapeutic benefit of inhibiting the different phases of the autophagic process remains a matter of debate (Mulcahy Levy and Thorburn, 2020). Here, we employed several classical or innovative pharmacological inhibitors of autophagy, acting at the initiation, nucleation, or completion phases of autophagy. We have interrogated the consequences for cell viability when these inhibitors are incubated under regular growing conditions. Despite some initial differences, at 12 h most of the assayed compounds inhibit the autophagic flux of MEF cells to a similar extent. Nonetheless, the evaluation of cell death evidences the cytotoxic effects of 3-methyladenine, SBI-0206965, Bafilomycin A_1_, and Chloroquine and the non-cytotoxic profile of MHY1485, Cpd18, SAR405, and Spautin-1. We examined the effects of these compounds on the cell viability of tumor-derived cell lines such as HCT116, HeLa, or SH-SY5Y and a non-tumoral embryonic cell line, HEK293. While most of the assayed inhibitors are non-cytotoxic, 3-methyladenine triggers a consistent and abrupt decrease of cell viability across all the cell lines. These results are in agreement with Eng and colleagues who have demonstrated that basal autophagy is dispensable for the survival of several cell lines in growing conditions. To impair autophagy, the authors employed genetic strategies targeting ATG7, ULK, or VPS34, or pharmacological approaches using the chloroquine analog, Lys01 (Eng et al., 2016). Collectively, these data support that the simple inhibition of basal autophagy is not cytotoxic for normal or tumor-derived cells in growing conditions. One possible explanation could be linked to the metabolic status of the cell. Notably, cells in growing conditions have access to a plethora of exogenous biomolecules necessary to fuel their metabolism, thus potentially compensating the impairment of the recycling pathways. In that sense, healthy metabolic cells with impaired basal autophagy would undergo cell demise if, a second, or even the same insult engages cytotoxic intracellular pathways. In agreement with this idea, our results show that some of the assayed autophagy inhibitors, for instance 3-methyladenine, SBI-0206965, Bafilomycin A_1_, and Chloroquine, while blocking basal autophagy, they also engage cell death. In this line, 3-methyladenine (Hou et al., 2012), SBI-0206965 (Tang et al., 2017), chloroquine (Eng et al., 2016), and Bafilomycin A_1_ (Yan et al., 2016) are reported inducers of deleterious cellular outcomes independently of their autophagy suppressive action. At the concentrations employed in this study, these compounds trigger cellular stresses that are able to initiate apoptotic intracellular pathways. Despite the cell protection conferred by q-VD-OPh, a subpopulation of cells still succumb to these inhibitors, pointing to the involvement of other subroutines of cell death. Notably, this situation is especially relevant in chloroquine-treated cultures since q-VD-OPh rescues less than 20% of the cells. These signaling pathways converge into the mitochondrion, as revealed by the resilience of *Bax−/− Bak−/−* double-knockout MEFs to undergo cell death. Our studies place the mitochondrion as the central target of the cytotoxic inhibitors of autophagy employed. According to this, those inhibitors of autophagy that are unable to stress the mitochondria would remain non-cytotoxic, regardless of their capacity to impair basal autophagy. This idea is also evidenced by employing *Fip200−/−* MEFs. Although these cells present a dramatic reduction of basal autophagy, the non-cytotoxic autophagy inhibitors remain non-cytotoxic, probably as a consequence of their inability to alter the mitochondrial homeostasis. On the opposite, autophagy deficient *Fip200−/−* MEFs are more vulnerable to cell death in response to compounds acting as disruptors of the mitochondrial viability, for instance 3-methyladenine and SBI-0206965. Basal or constitutive autophagy is active in healthy cells cultured in growing conditions and contributes to the preservation of cellular homeostasis. On the other hand, inducible autophagy is a cellular stress-responsive adaptation to unfavorable environmental conditions. In this sense, chemo- and radiotherapy are stressful stimuli that elicit inducible adaptative autophagy (Nagelkerke et al., 2015). Our results support that starvation-induced autophagy is not qualitatively superior than basal autophagy at protecting cells from staurosporine-elicited cell death (results not shown). In other words, inducible autophagy would be insufficient to counteract staurosporine-mediated mitochondrial damage of cells under adverse metabolic conditions (*e.g.*, starved cells). The molecular clues that differentiate basal and inducible autophagy, if any, are not yet well characterized. Based on our own data and the more recent literature, we surmise both types of autophagy are part of the same continuum (“the basal-to-inducible autophagy continuum”), only differentiated by their intensity.

3-methyladenine, the main subject of this study, is a compound identical to the DNA adduct N3-methyl-adenine (3meA). The DNA content of 3meA adducts increases in response to metabolic intermediates (*i.e.*, S-adenosyl-methionine) or alkylating poisons (Rydberg and Lindahl, 1982). 3meA adduct, which projects into the minor groove of the DNA, is a potent inhibitor of both RNA and most DNA polymerases and accounts for most of the cytotoxicity in response to alkylating drugs (Engelward et al., 1998; Johnson et al., 2007). The collapse of unstable blocked replication forks provokes double-stranded DNA breaks (DSBs) (Bobola et al., 2012) and replication stress (Burhans and Weinberger, 2007). The phosphorylation of histone H2A.X on Ser139 (*γ*-H2A.X) is a widely accepted marker of DSB (Rogakou et al., 1998). In our experiments, cytotoxic concentrations of 3-methyladenine trigger the most prominent induction of *γ*-H2A.X compared to the other cytotoxic inhibitors of autophagy. This genotoxicity is not a direct consequence of caspase activation since the addition of q-VD-OPh to the culture media is unable to reduce the burst of 3-methyladenine-elicited *γ*-H2A.X. These results raise interesting questions about the mechanism of 3-methyladenine-mediated genotoxicity. It is tempting to attribute this genotoxicity to the direct incorporation of 3-methyladenine into the replicating DNA. However, 3-methyladenine is an ambident nucleophile, and methylation of DNA requires electrophilic compounds (Wu et al., 2013). In this sense, *in vitro* experiments evidence the inefficiency of 3-methyladenine at directly generating DNA breaks into plasmid DNA (J. Ribas & V.J. Yuste, unshown data). One alternative explanation could be related to the metabolic reprogramming exerted by this drug. Strikingly, 3-methyladenine rewires the carbohydrate metabolism resulting in an accumulation of glucose-6-phosphate, fructose-6-phosphate and phosphoenol pyruvate. This phenomenon is caused by cAMP and is unrelated to its intrinsic autophagy inhibitory function (Caro et al., 1988). In this sense, it is known that reducing sugars (such as glucose-6-phosphate or fructose-6-phosphate) can modify nucleic acids in a non-enzymatic reaction. Indeed, glucose-6-phosphate and fructose-6-phosphate trigger DNA damage, mutagenesis, and cytotoxicity (Bucala et al., 1985; Levi and Werman, 2003). Based on our results and the broad usage of 3-methyladenine, it would be of outstanding interest to go deeply into the molecular mechanisms of 3-methyladenine generating DNA damage and its relationship with metabolic changes.

With regard to the protective role of FIP200-mediated autophagy facing genotoxicity, we found that the upregulation of *γ*-H2A.X in response to 3-methyladenine is enhanced in the autophagy-deficient *Fip200−/−* MEFs. However, the simple inhibition of autophagy (either with SAR405 or in *Fip200−*/*−* MEFs) is unable to increase *γ*-H2A.X. These data indicate that increased *γ*-H2A.X is an autophagy-independent effect of 3-methyladenine and that autophagy is counteracting this genotoxic effects. This is in agreement with the role of autophagy in regulating DNA repair (Hewitt and Korolchuk, 2017). Notably, FIP200 deficiency diminishes DNA repair in response to irradiation, camptothecin or etoposide in a p62-dependent manner, thus increasing the cytotoxicity mediated by these stimuli (Bae and Guan, 2011). In support of these data, a direct link between p62 and the DNA damage machinery was established through the identification of its inhibitory role over RNF168, an H2A ubiquitinase that is essential for the recruitment of multiple DNA repair proteins (Wang et al., 2016). Moreover, autophagy is increased in cells subjected to an intense DNA damage in response to genotoxic stressors and is necessary for the recovery from replication stress (Vanzo et al., 2020).

Autophagy is known to counteract oncogenic transformation of normal cells but, at the same time, it promotes tumor progression and resistance of tumor-derived cells (Galluzzi et al., 2017). For this reason, the inhibition of autophagy is under consideration as a sensitizing mechanism towards chemotherapeutic agents. Our results evidence that the cytotoxic inhibitors of autophagy promote cytotoxicity and increase DNA damage. The degree of genomic damage could derive into diverse consequences: first, the engagement of deleterious intracellular pathways if levels of DNA damage surpass the cellular DNA repair capacity; or, second, the survival of cells able to repair the inflicted DNA damage. In this last scenario, tumor cells could accumulate genomic mutations, thus potentially worsening their malignant behavior. Among the cytotoxic inhibitors of autophagy studied here, 3-methyladenine is the top inducer of DNA damage. This compound shows unfavorable features to be translated into the clinics since its poor hydrosolubility and effective concentrations in the millimolar range (Seglen and Gordon, 1982; Petiot et al., 2000; Boya et al., 2005; Wu et al., 2010; Devereaux et al., 2013; Wu et al., 2013). However, taking into consideration that autophagy also participates in the evasion of tumor cells from immune surveillance, the interest of targeting VPS34 in the field of cancer immunotherapy is reinforced (Mgrditchian et al., 2017; Noman et al., 2020). Indeed, the inhibition of VPS34 is under consideration as an adjuvant therapy for the inhibitors of the immune checkpoints (ICIs). SAR405, or the new inhibitor of VPS34 SB02024, trigger an inflammatory profile that allows the infiltration of antitumoral immune cells to the tumor bed (Noman et al., 2020). Besides SAR405, our research identifies MHY1485 and Spautin-1 as interesting pharmacological tools in terms of their efficacy in blocking autophagy and their lack of cytotoxicity. In this sense, our studies support the interest of these drugs as safe and efficient non-cytotoxic inhibitors of autophagy to potentially be applied in new therapeutic strategies.

Overall, 3-methyladenine-driven DNA damage and cytotoxicity call for acautionary usage of this drug in those experimental paradigms where cell survival/death is interrogated. On the other hand, the lack of cytotoxicity of SAR405, MHY1485, and Spautin-1 portrays these compounds as great tools to address the role of autophagy in cell survival/death decisions, without the interference of distracting cytotoxic off-target effects.

## Data Availability Statement

All datasets generated for this study are included in the article/**Supplementary Material**.

## Author Contributions

JC performed the experiments and participated in the elaboration of the graphs. VY contributed to the conceptualization of the research, provided some resources, and helped in writing the first draft as well as the final manuscript. JB contributed to the elaboration of the graphs, writing the first draft, and provided some equipment and resources. JR conceived and designed the research, performed the formal analysis, validated the results, supervised the research, contributed to writing the first draft, and the final draft. JR provided the main resources and funding for this research. All authors contributed to the article and approved the submitted version.

## Funding

This work and its publication fee were supported by a “Retos” project (SAF2016-78657-R) from the “Ministerio de Economía, Industria y Competitividad”/Fondos FEDER (Spain), granted to JR. JR is a Serra Húnter Fellow. JC holds a Ph.D. student fellowship from University of Lleida.

## Conflict of Interest

The authors declare that the research was conducted in the absence of any commercial or financial relationships that could be construed as a potential conflict of interest.

## Supplementary Material

Supplementary Figure 1 Quantification of monodansylcadaverine-positive vesicles per cell. MEFs were treated for 6h with the autophagy inhibitory drugs employed in this study. After staining with Monodansylcadaverine (MDC), images from Figure 1 were analyzed using ImageJ software. Plots are the result of counting MDC-positive vesicles of at least 50 cells per condition. Bar value is the mean ± SEM. Student’s t-test **P<0.005 and ***P<0.001.

Supplementary Figure 2 Cell death by 3-methyladenine, SBI-0206965, Bafilomycin A1 and Chloroquine in the presence or absence of q-QVD-OPh. MEFs were treated with the drugs at the concentrations stated in the figure. A) After 36h, pictures using a phase contrast microscope were obtained. Scale bar: 50 μm. B) After 24h and 48h, the percentage of propidium iodide (PI)-positive cells (dead cells) was determined by flow cytometry. Bar value is the mean ± SEM (n=3). Student’s t-test ***P<0.001.

Supplementary Figure 3 3-methyladenine or Cpd18 inhibit the autophagic flux. A) Western blot of protein extracts from MEFs treated for 8h with growing concentrations of 3-MA or Cpd18 in the presence (+) or absence (-) of 100 nM BafA1. Intensity of the p62 band, normalized to the loading of each lane (Naphtol Blue (NB) staining) and referred to “0” with BafA1, was shown. The shown Western blot is a representative one out of 3 independent experiments. MEFs were loaded with DALGreen reagent for 30 min before subjecting them to fresh cell culture media (Control) or media containing 10 mM 3-MA, 0.5 mM Cpd18, 50 μM Chloroquine or 100 nM BafA1 for 8h. B) Fluorescence images were obtained using an inverted microscope. Scale bar: 50 μm. Representative images are shown. C) Quantification of the “Averaged DALGreen Intensity/cell” from at least 50 cells from three independent wells. Fluorescence is expressed in “arbitrary units of fluorescence” (a.u.f.). Bar value is the mean ± SEM. Student’s t-test ***P<0.001.

Supplementary Figure 4 3-methyladenine upregulates g-H2A.X independently of the inhibition of caspase-3. Protein extracts of MEFs untreated (Control) or 10 mM 3-MA treated for 24h in the presence or absence of 40 μM of q-VD-OPh (QVD), were analyzed by western blot. The antibodies used were anti-caspase-3, anti-g-H2A.X and anti-H2A.X. The membrane stained with Naphtol Blue (NB) served as a loading control. The images are one representative Western blot out of three independent experiments.

## Abbreviations

3-MA, 3-methyladenine; 3meA, 3-methyladenine adducts; AB, alamarBlue; ATG, autophagy-related gene; AVOs, acidic vesicular organelles; BafA_1_, Bafilomycin A_1_; CQ, Chloroquine; cyt C cytochrome C; DEVD, Ac-Asp-Glu-Val-Asp-AFC; DKO, double knockout; DSBs, double strand breaks; ER, endoplasmatic Reticulum; FIP200, Focal adhesion Kinase family interacting protein of 200 kDa; GA, Golgi apparatus; LC3, microtubule-associated protein 1 light chain 3; MDC, monodansylcadaverine; MEFs, mouse embryonic fibroblasts; MHY, MHY1485; mTOR, mammalian target of Rapamycine; NB, Naphthol blue; p62, p62/SQSTM1; PI, propidium iodide; PI3K, phosphatidylinositol 3-kinase; q-VD-OPh, N-(2-Quinolyl)valyl-aspartyl-(2,6-difluorophenoxy)methyl ketone; SAR, SAR405; SBI, SBI-0206965; SPT-1, Spautin-1; STS, staurosporine; SSBs, single strand breaks; ULK, Unc-51-like kinase; USP, ubiquitin-specific peptidase; V-ATPase, vacuolar-type H^+^-V-ATPase; VPS34, vacuolar protein sorting 34; Wn, Wortmannin.
