# Supplementary figures and images for: Cell Death Triggered by the Autophagy Inhibitory Drug 3-Methyladenine in Growing Conditions Proceeds With DNA Damage
Source: Front Pharmacol. 2020 Oct 15;11:580343. doi: 10.3389/fphar.2020.580343 (PMC7593545; doi:10.3389/fphar.2020.580343)

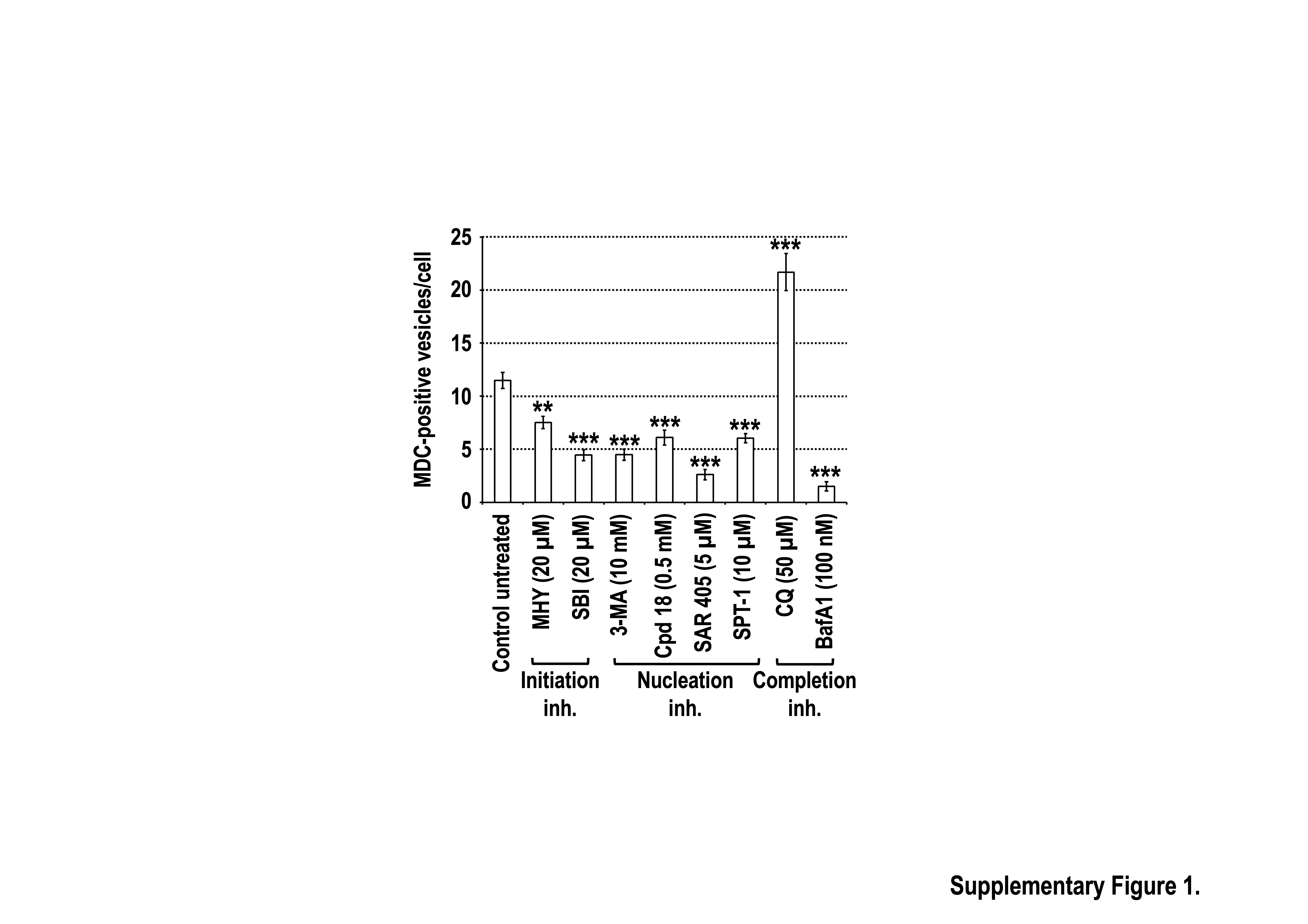

Supplement: Supplementary Figure 1 — Quantification of monodansylcadaverine-positive vesicles per cell. MEFs were treated for 6h with the autophagy inhibitory drugs employed in this study. After staining with Monodansylcadaverine (MDC), images from Figure 1 were analyzed using ImageJ software. Plots are the result of counting MDC-positive vesicles of at least 50 cells per condition. Bar value is the mean ± SEM. Student’s t-test **P<0.005 and ***P<0.001. [file Image_1.jpeg]

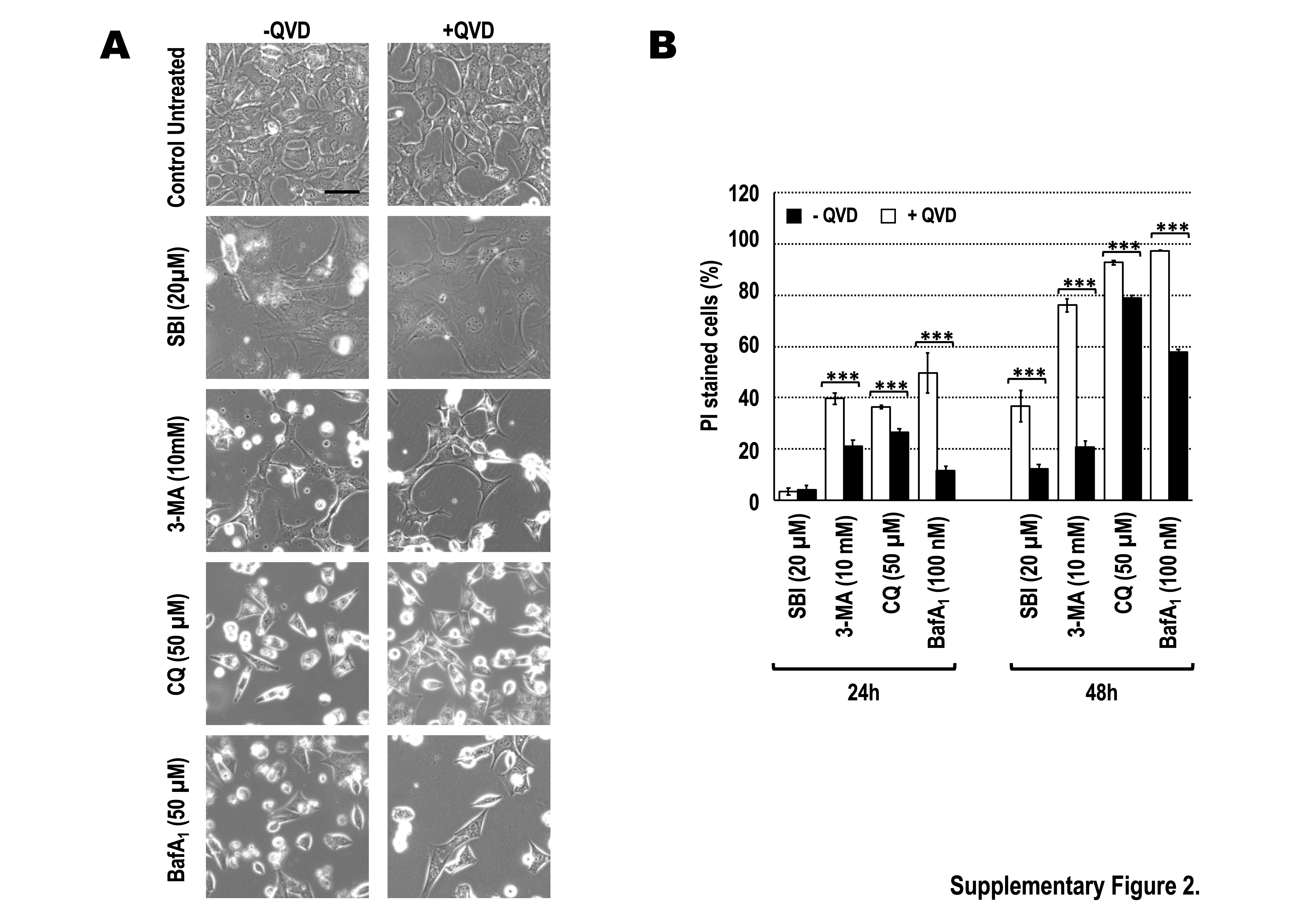

Supplement: Supplementary Figure 2 — Cell death by 3-methyladenine, SBI-0206965, Bafilomycin A1 and Chloroquine in the presence or absence of q-QVD-OPh. MEFs were treated with the drugs at the concentrations stated in the figure. A) After 36h, pictures using a phase contrast microscope were obtained. Scale bar: 50 μm. B) After 24h and 48h, the percentage of propidium iodide (PI)-positive cells (dead cells) was determined by flow cytometry. Bar value is the mean ± SEM (n=3). Student’s t-test ***P<0.001. [file Image_2.jpeg]

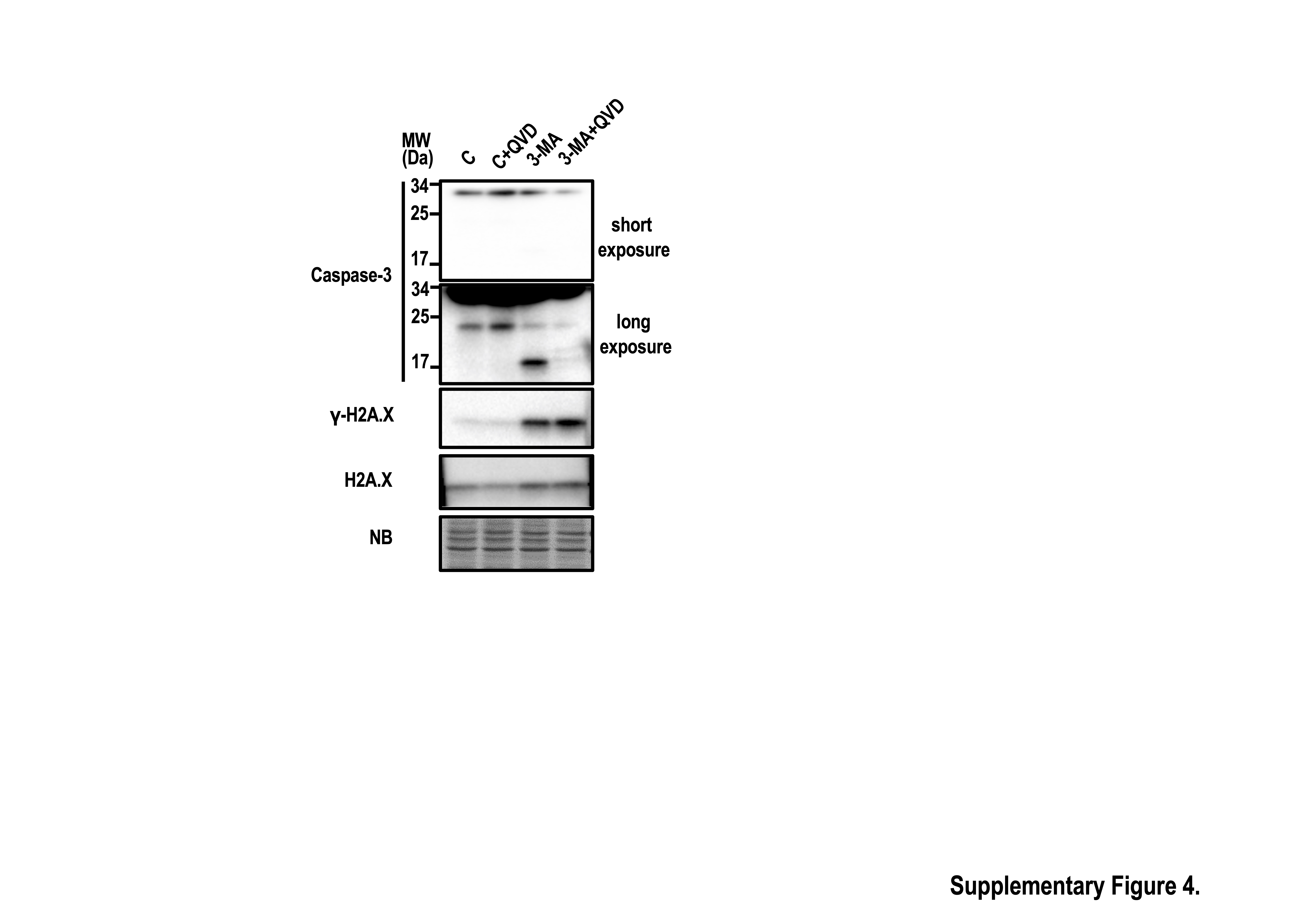

Supplement: Supplementary Figure 4 — 3-methyladenine upregulates g-H2A.X independently of the inhibition of caspase-3. Protein extracts of MEFs untreated (Control) or 10 mM 3-MA treated for 24h in the presence or absence of 40 μM of q-VD-OPh (QVD), were analyzed by western blot. The antibodies used were anti-caspase-3, anti-g-H2A.X and anti-H2A.X. The membrane stained with Naphtol Blue (NB) served as a loading control. The images are one representative Western blot out of three independent experiments. [file Image_4.jpeg]
